# *Photobacterium damselae* subsp. *damselae*, a bacterium pathogenic for marine animals and humans

**DOI:** 10.3389/fmicb.2013.00283

**Published:** 2013-09-25

**Authors:** Amable J. Rivas, Manuel L. Lemos, Carlos R. Osorio

**Affiliations:** Institute of Aquaculture, University of Santiago de CompostelaSantiago de Compostela, Spain

**Keywords:** *Photobacterium damselae*, hemolysin, damselysin, *hlyA*, pore-forming toxin

## Abstract

*Photobacterium damselae* subsp. *damselae* (formerly *Vibrio damsela*) is a pathogen of a variety of marine animals including fish, crustaceans, molluscs, and cetaceans. In humans, it can cause opportunistic infections that may evolve into necrotizing fasciitis with fatal outcome. Although the genetic basis of virulence in this bacterium is not completely elucidated, recent findings demonstrate that the phospholipase-D Dly (damselysin) and the pore-forming toxins HlyA_pl_ and HlyA_ch_ play a main role in virulence for homeotherms and poikilotherms. The acquisition of the virulence plasmid pPHDD1 that encodes Dly and HlyA_pl_ has likely constituted a main driving force in the evolution of a highly hemolytic lineage within the subspecies. Interestingly, strains that naturally lack pPHDD1 show a strong pathogenic potential for a variety of fish species, indicating the existence of yet uncharacterized virulence factors. Future and deep analysis of the complete genome sequence of *Photobacterium damselae* subsp. *damselae* will surely provide a clearer picture of the virulence factors employed by this bacterium to cause disease in such a varied range of hosts.

## *PHOTOBACTERIUM DAMSELAE* SUBSP. *DAMSELAE*

*Photobacterium damselae* subsp. *damselae* is a marine bacterium of the family *Vibrionaceae* that causes infections in a variety of marine animals and also in humans. A bit of historic perspective is necessary in order to understand its current taxonomic placement as well as the changes in its nomenclature during the past decades. In 1971 an “unnamed marine *Vibrio*” was isolated as the causative agent of a human infectious case (Morris et al., 1982)*. *Later, this same organism was isolated from skin ulcers of damselfish (*Chromis punctipinnis*) and the name *Vibrio damsela* was first coined (Love et al., 1981). Further genetic and phenotypic studies indicated that the strains of *V. damsela* were closely related to species of the genus *Photobacterium*, and the name *Photobacterium damsela* was proposed (Smith et al., 1991). In 1995, DNA–DNA hybridization data and 16S rRNA sequence analysis demonstrated that *Photobacterium damsela* was closely related to a fish pathogen formerly named *Pasteurella piscicida*, the causative agent of pasteurellosis in fish. Hence, these two organisms were assigned to the same species epithet, *Photobacterium damselae*, with category of subspecies (Gauthier et al., 1995), *Photobacterium damselae* subsp. *damselae *and *Photobacterium damselae* subsp. *piscicida* respectively. Despite their similarity at the 16S gene sequence and the high percentage of DNA–DNA relatedness between them, these two subspecies are clearly distinguished by several phenotypical traits (Fouz et al., 1992; Magariños et al., 1992; Thyssen et al., 1998; Botella et al., 2002). Differential phenotypical tests of interest for subspecies discrimination that are positive only for subsp. *damselae* include motility, nitrate reduction and hemolysis on sheep blood agar. Of special relevance is the ability of most subsp. *damselae* strains to grow at 37°C (a temperature inhibitory for subsp. *piscicida*), a trait that allows *Photobacterium damselae* subsp. *damselae* to potentially colonize and establish an infection in a homeotherm animal.

### A PATHOGEN OF MARINE ANIMALS

*Photobacterium* *damselae* subsp. *damselae* is an autochthonous member of aquatic ecosystems. Strains of this pathogen have been isolated from sea and estuarine waters, from seaweeds, from apparently uninfected marine animals (Buck et al., 2006; Serracca et al., 2011) and from seafood (Lozano-León et al., 2003; Chiu et al., 2013), and it is considered a common member of the natural microbiota of healthy carcharhinid sharks (Grimes et al., 1985).

In addition, *Photobacterium damselae* subsp. *damselae* is considered a primary pathogen of several species of wild fish (damselfish, catfish, shark, stingray, etc.), as well as of fish species of economical importance in aquaculture, causing wound infections and hemorrhagic septicemia. Cultivated species reported to be affected by this pathogen include turbot (*Psetta maxima*; Fouz et al., 1992), rainbow trout (*Oncorhynchus mykiss*; Pedersen et al., 2009), ovate pompano (*Trachinotus ovatus*; Zhao et al., 2009), eel (*Anguilla reinhardtii*; Ketterer and Eaves, 1992), sea bream (*Sparus aurata*; Vera et al., 1991), sea bass (*Dicentrarchus labrax*), yellowtail (*Seriola* *quinqueradiata*), redbanded seabream (*Pagrus auriga*), white seabream (*Diplodus sargus*), and meagre (*Argyrosomus regius*; Labella et al., 2006, 2010a, b), among others. The recent first reports on isolation of this pathogen from diseased marine fish of new cultured species, suggest that *Photobacterium damselae* subsp. *damselae* can be considered as an emerging pathogen in marine aquaculture (Labella et al., 2011).

Moreover, *Photobacterium damselae* subsp. *damselae* has been isolated as a pathogen of brown shark (*Carcharhinus plumbeus*; Grimes et al., 1984), of reptiles as the leatherback sea turtle (*Dermochelys coriacea*; Obendorf et al., 1987), molluscs (*Octopus joubini*; Hanlon et al., 1984), crustaceans (Song et al., 1993; Vaseeharan et al., 2007), dolphins (*Tursiops truncatus* and *Delphinus delphis*; Fujioka et al., 1988; Buck et al., 1991) and Bryde’s whale (*Balaenoptera edeni*; Buck et al., 1991).

Virulent isolates are capable of survival in seawater microcosms at 14–22°C as culturable bacteria for long periods of time, maintaining their infectivity for fish (Fouz et al., 1998). Similarly, this pathogen can infect new fish hosts through water, and the spread of the disease depends largely on water temperature and salinity (Fouz et al., 2000). Typical signs of the disease in infected fish include hemorrhaged areas on the body surface and ulcerative lesions. In damselfish, ulcers typically occur in the region of the pectoral fin and caudal peduncle and may reach 5–20 mm in diameter (Love et al., 1981), while in turbot the most remarkable symptoms are extensive hemorrhages in eyes, mouth, and jaws (Fouz et al., 1995).

Experimental inoculation of *Photobacterium damselae* subsp. *damselae* extracellular products (ECPs) in a redbanded seabream model was reported to cause lethargy, increase in the respiratory frequency, mucus production, presence of ascitic liquid, hemorrhagic and enlarged liver, and hemorrhages in the abdominal cavity (Labella et al., 2010b). A histological analysis of internal organs in experimentally infected turbot indicated that the ECPs and cells of virulent strains cause similar tissue damage (Fouz et al., 1995). Structural changes included destruction and necrosis of cells, as well as accumulation of blood cells in interstitial tissue.

### *Photobacterium damselae* subsp. *damselae* AS A HUMAN PATHOGEN

Most of the reported infections caused by *Photobacterium damselae* subsp. *damselae* in humans have their primary origin in wounds exposed to salt or brackish water, inflicted during fish and tools handling (Morris et al., 1982; Dryden et al., 1989; Yuen et al., 1993; Shin et al., 1996; Tang and Wong, 1999; Barber and Swygert, 2000; Goodell et al., 2004; Aigbivhalu and Maraqa, 2009). Unusual cases of infection after ingestion of raw seafood (Kim et al., 2009) and through the urinary tract by exposure to sea water (Alvarez et al., 2006) were also reported. The majority of the cases occurred in coastal areas of the United States of America, Australia, and Japan.

*Photobacterium damselae* subsp. *damselae* can cause an extreme variant of a highly severe necrotizing fasciitis, and antibiotic administration proved unable to control the progression of fatal infections in some cases (Clarridge and Zighelboimdaum, 1985; Fraser et al., 1997; Yamane et al., 2004). It is interesting to note that some authors recommend to surgically debride and amputate without hesitation at a very early point of the infection by *Photobacterium damselae* subsp. *damselae*, to save the lives of patients (Goodell et al., 2004). Some patients infected by *Photobacterium damselae* subsp. *damselae* developed multiple organ failure within a few hours from the onset of initial symptoms, despite intensive chemotherapy and surgical treatments. As an example, in a fatal case reported in 1984 in which a patient injured his hand while handling a catfish, bulle formation occurred on the hand and a marked edema extended through the forearm in less than 24 h (Clarridge and Zighelboimdaum, 1985), and although the affected area was extensively debrided the patient died after a series of complications. The bacterium was recovered in high numbers from the tissue sample but only in very small numbers from the bulle fluid. In another fatal case reported in a patient injured while handling fish, *Photobacterium damselae* subsp. *damselae* was isolated in pure culture from wound specimens but failed to be isolated from blood samples (Fraser et al., 1997). These observations prompted these authors to suggest that a virulence factor or systemic toxin released by this bacterium contributed to the tissue damage and to the fatal outcome, rather than the septicemia itself. However, in other clinical cases this pathogen was recovered from blood (Perez-Tirse et al., 1993; Shin et al., 1996; Yamane et al., 2004).

Necrotizing fasciitis due to *Photobacterium damselae* subsp. *damselae* demonstrates more serious complications and a higher mortality rate than that caused by *Vibrio vulnificus*. While *V. vulnificus* usually affects persons with underlying diseases (as chronic liver disease and diabetes mellitus), necrotizing fasciitis by *Photobacterium damselae* subsp. *damselae* sometimes occurs in healthy hosts (Morris et al., 1982; Perez-Tirse et al., 1993; Yuen et al., 1993).

### VIRULENCE FACTORS

#### Iron uptake systems

Early studies reported that *Photobacterium damselae* subsp. *damselae* can utilize heme, hemoglobin and ferric ammonium citrate as sole iron sources *in vitro* (Fouz et al., 1994). The complete sequence of 10 genes encoding a system for the utilization of heme as iron source was described in a human isolate of *Photobacterium damselae* subsp. *damselae*, and cloning of the complete system into *E. coli* conferred to this species the ability to use hemin and hemoglobin as iron sources (Rio et al., 2005). The presence of the heme receptor gene *hutA* was demonstrated in subsp. *damselae* isolates from fish and humans, and the identity at the DNA sequence level between the heme uptake clusters of subsp. *damselae* and subsp. *piscicida* strains was 97% (Rio et al., 2005). Although no functional studies were conducted with the heme uptake genes of subsp. *damselae*, it was recently demonstrated that this cluster is essential for heme utilization in subsp. *piscicida*, and two genes of a hemin ABC-transporter proved to be expressed during the infective process in a fish model (Osorio et al., 2010). Actually, an increase in the susceptibility of both fish and mice to infection by virulent *Photobacterium damselae* subsp. *damselae* strains in virulence assays conducted with iron-overloaded animals had been demonstrated in former studies (Fouz et al., 1994). It is also known that this bacterium produces a hydroxamate-type siderophore, and the synthesis of several high-molecular weight outer membrane proteins induced under iron limitation conditions was reported (Fouz et al., 1997). Although the precise chemical structure of the siderophore(s) is so far unknown, recent unpublished work from our laboratory demonstrated that vibrioferrin is being produced by some strains.

#### Cytotoxins with hemolytic activity

Pioneering studies (Kreger, 1984) reported the existence of a correlation between the ability of *Photobacterium damselae* subsp. *damselae* isolates to cause disease in mice and the production of large amounts of a heat-labile cytolytic toxin* in vitro*. Later, the same authors purified a toxin that exhibited strong hemolytic activity against erythrocytes of a variety of animal species (Kothary and Kreger, 1985). In subsequent studies, this toxin named damselysin (Dly) was defined as a phospholipase-D active against sphingomyelin, with hemolytic activity (Kreger et al., 1987), and its gene (*dly*) was cloned and sequenced (Cutter and Kreger, 1990). Dly was thus considered to be the main virulence factor of *Photobacterium damselae* subsp. *damselae* for mice. Further studies reported the existence of hemolytic strains of *Photobacterium damselae* subsp. *damselae* that tested negative for *dly* gene, which suggested that Dly was not the only hemolysin in the subspecies (Osorio et al., 2000). It was also demonstrated by several authors that presence of *dly* is not a prerequisite for the hemolytic activity and for the pathogenicity for mice or fish, since *dly* negative strains bear virulence potential for animals and also toxicity for homeotherm and poikilotherm cell lines (Osorio et al., 2000; Labella et al., 2010b).

**FIGURE 1:**
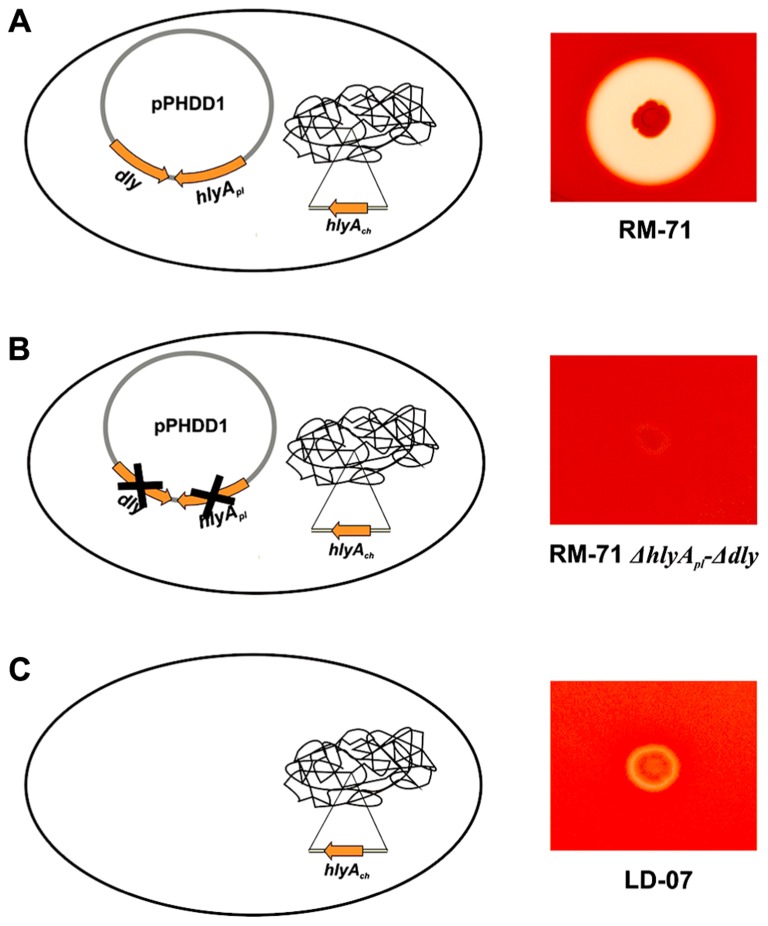
**Hemolytic phenotypes in sheep blood agar of *Photobacterium damselae* subsp. *damselae* strains. (A)** strains that naturally harbor pPHDD1 plasmid produce the three hemolysins Dly (damselysin), HlyA_pl_, and HlyA_ch_ (this last one, encoded in the chromosome), and cause a wide hemolytic halo in sheep blood agar. **(B)** a double mutant for *dly* and *hlyA*_pl_ genes shows a weak hemolytic phenotype, similar to that of a naturally plasmidless strain **(C)**, that only produces HlyA_ch_.

The genomic context of *dly* gene remained uncharacterized for decades, and it was initially proposed that it could be carried on a mobile or unstable DNA element (Cutter and Kreger, 1990). Recently, the authors’ laboratory identified and characterized a 150 kb plasmid, which was dubbed pPHDD1, that contains the genes for Dly as well as for a HlyA toxin of the pore-forming toxin family (Rivas et al., 2011). Only a fraction of *Photobacterium damselae* subsp. *damselae* strains harbor pPHDD1, and these strains exhibit a much wider hemolytic halo on sheep blood agar plates than the plasmidless strains (**Figure 1**). Interestingly, pPHDD1 occurs in both fish and human isolates and it is not restricted to a unique animal host species (Rivas et al., 2011). In addition to being necessary to cause strong hemolytic haloes on blood agar plates, the two pPHDD1-encoded hemolysins play a crucial role in virulence for fish and mice in strains that naturally harbor the plasmid. Hence, mutation of both *dly* and *hlyA* genes in a pPHDD1-harboring strain renders the strain non-virulent for fish, and only slightly virulent for mice (**Table 1**), and the hemolytic phenotype on sheep blood agar of a *dly* and *hlyA* double mutant resembles that of naturally plasmidless strains (**Figure 1**; Rivas et al., 2011, 2013).

**Table 1:** Role of the three *Photobacterium damselae* subsp. *damselae* hemolysins in virulence for mice and fish (turbot).

Strain	Hemolysin(s) produced	Number of dead mice (*n* = 15)	Number of dead fish (*n* = 15)
Parental	HlyA_ch_ HlyA_pl_ Dly	15	15
*ΔhlyA*_ch_	HlyA_pl_ Dly	13	9*
*ΔhlyA*_pl_	HlyA_ch_ Dly	10*	14
*Δdly*	HlyA_ch_ HlyA_pl_	10*	4*
*ΔhlyA*_ch _ΔhlyA_pl _	Dly	12	5*
*ΔhlyA*_ch _Δdly	HlyA_pl_	9*	0*
*ΔhlyA*_pl _Δdly	HlyA_ch_	3*	0*
*ΔhlyA*_ch _ΔhlyA_pl _Δdly	none	0*	0*

Parental and mutant strains were inoculated in groups of 15 animals, at doses of 2.1 × 10^*6*^ bacterial cells per mouse and 2.1 × 10^*4*^ bacterial cells per fish. The number of dead animals out of the total number of inoculated animals (15) is indicated. Asterisks denote that significant differences exist between a given mutant and the parental strain, using U-test (*P < 0.05).

The hemolytic activity exhibited by plasmidless strains was recently demonstrated to be caused by a chromosome-encoded *hlyA* gene, which was dubbed *hlyA*_ch_ in order to differentiate it from the plasmid *hlyA* gene (hereafter *hlyA*_pl_; Rivas et al., 2013). It was found that all the hemolytic *Photobacterium damselae* subsp. *damselae* strains harbor *hlyA*_ch_ gene, which is the only hemolytic determinant in plasmidless strains. Thus, pPHDD1-harboring isolates produce three different hemolysins. In hemolytic assays carried out with bacterial ECPs and with sheep erythrocytes, it was demonstrated that Dly acts in a synergistic manner with HlyA_pl_ and HlyA_ch_, whereas the effect between HlyA_pl_ and HlyA_ch_ showed to be additive but not synergistic (Rivas et al., 2013).

Although each of the three hemolysins individually proved to be sufficient to cause death in mice, each one contributes to virulence in a different degree. The contribution of HlyA_ch _to virulence for mice is the lowest among the three toxins. Altogether, albeit the highest values of mortality for mice are achieved only when the three hemolysins are being produced, Dly and HlyA_pl_ demonstrated to be main contributors in the virulence of *Photobacterium damselae* subsp. *damselae* for mice (Rivas et al., 2013; **Table 1**).

Interestingly, the contribution of each hemolysin to virulence was found to vary depending on whether the host animal tested was mouse or turbot. When virulence experiments were conducted with turbot, it was found that among all the hemolysin gene mutants only the Dly-producing strains caused death in fish. This finding demonstrated that any of the two HlyA alone does not cause death in turbot, but rather one of the two HlyA needs the presence of either Dly or the other HlyA to cause death in fish. The production of Dly in combination with any of the two HlyA caused an increase in the number of dead fish with respect to the production of Dly alone, and this increase was found to be particularly evident when Dly was combined with HlyA_ch_. This clearly suggests that, unlike what is observed in mice, the contribution of hemolysins to virulence for fish is not so much based on the individual effects of each hemolysin but rather on the combined (synergistic) effects between Dly and HlyA (Rivas et al., 2013; **Table 1**). These findings also state the importance of pPHDD1 plasmid in virulence for fish, since Dly is necessary for the synergistic effect.

#### Other exoenzymes and exotoxins: toxicity of the extracellular products

Early studies detected several enzymatic activities in the ECPs of *Photobacterium damselae* subsp. *damselae*, which included phospholipase and hemolysin activities (Fouz et al., 1993). More recent data confirmed that the ECPs of *Photobacterium damselae* subsp. *damselae* are strongly lethal for fish, and enzymatic activities such as amylase, lipase, phospholipase, alkaline phosphatase, esterase–lipase, acid phosphatase, and β -glucosaminidase were evidenced (Labella et al., 2010b). Moreover, treatment at 100°C for 10 min of the ECPs abolished the ability to cause death in fish, suggesting that toxicity was not due to the thermorresistant lipopolysaccharide content. *Photobacterium damselae *subsp. *damselae *ECPs also displayed cytotoxic activity for different fish and mammalian cell lines (Wang et al., 1998; Labella et al., 2010b). Different studies found a correlation between virulence of the strain and toxicity of the ECPs, with toxicity being limited to ECPs from strains that were also virulent for fish (Fouz et al., 1995; Labella et al., 2010b). Of maximum interest is the observation that strains lacking pPHDD1 plasmid and thus being negative for *dly* and *hlyA*_pl_ genes, are virulent for fish and their ECPs are cytotoxic for cell lines. In addition, a comprehensive study reported that none of the enzymatic activities detected in the *Photobacterium damselae* subsp. *damselae* ECPs could be related with the degree of toxicity either *in vivo *or *in vitro* (Labella et al., 2010b). Most *Photobacterium damselae* subsp. *damselae* strains test negative for protease activities as caseinase and gelatinase (Fouz et al., 1992; Labella et al., 2010b). This suggests that other, yet uncharacterized molecules produced by *Photobacterium damselae* subsp. *damselae* cells play a role in toxicity for animals and for cell lines. In this regard, previous studies detected the existence of an acetylcholinesterase activity (ictiotoxin) with neurotoxic activity in several species of *Vibrionaceae*, including *Photobacterium damselae *subsp. *damselae* (Perez et al., 1998), although the genetic basis for this neurotoxic activity remains unknown.

### FUTURE PERSPECTIVES

An interesting observation that remains to be explained at the genetic level is the finding that plasmidless *Photobacterium damselae* subsp. *damselae* strains are virulent for fish and toxic for homeotherm and poikilotherm cell lines (Fouz et al., 1993; Osorio et al., 2000; Labella et al., 2010b, 2011). Since plasmidless strains lack *dly* and *hlyA*_pl_ genes, and since *dly hlyA*_pl_ double mutants are significantly reduced in its virulence for both mice and fish (Rivas et al., 2013), it is evident that plasmidless strains encode virulence factors that either are not encoded by pPHDD1-harboring strains or their expression is repressed in presence of pPHDD1-encoded genes.

The recent completion of the genome sequence of the type strain (ATCC 33539) of this subspecies (deposited in GenBank database in several separate contigs, under accession number ADBS00000000), allows an *in silico* analysis to search for candidate genes encoding potential toxins and other virulence factors. The type strain harbors pPHDD1 plasmid, and preliminary analyses also indicated the presence of genes encoding a type III hemolysin (open reading frame number: VDA003208), and a putative murine toxin (VDA000322) among others. The existence of yet uncharacterized plasmids is also evidenced in the complete genome of ATCC 33539. Studies to functionally characterize novel plasmid content and candidate virulence genes of *Photobacterium damselae* subsp. *damselae* strains are currently under way. It is expected that a deep analysis of the complete genome sequence of *Photobacterium damselae* subsp. *damselae* strains with different isolation origins and virulence properties will provide a clearer picture of the virulence factors employed by this bacterium to cause disease in such a varied range of hosts.

## Conflict of Interest Statement

The authors declare that the research was conducted in the absence of any commercial or financial relationships that could be construed as a potential conflict of interest.
